# Cardiopulmonary exercise test combined with red blood cell distribution width to predict cardiovascular complication of thoracic surgery

**DOI:** 10.1038/s41598-024-54220-8

**Published:** 2024-02-15

**Authors:** Quanqiang Lin, Qingheng Zhao, Qiang Xiao, Yuanmin Li

**Affiliations:** 1 Department of Cardiology, The Second Affiliated Hospital, Shandong First Medical University & Shandong Academy of Medical Sciences, No.366 Taishan Street, Taian, 271000 People’s Republic of China; 2 Department of Cardiology, Linyi People’s Hospital, Linyi, People’s Republic of China

**Keywords:** Cardiopulmonary exercise test, Red blood cell distribution width, Thoracic surgery, Cardiovascular complications, Cardiology, Diseases, Health care, Medical research

## Abstract

Cardiovascular complications in patients undergoing thoracic surgery, which physicians have a limited ability to predict, are often unavoidable and resulting in adverse outcome. Cardiopulmonary exercise testing (CPET), the gold standard of cardiopulmonary function evaluation, has also been proved to be a preoperative risk assessment tool. Meanwhile, elevated red blood cell distribution width (RDW) has surged as a biochemical marker in the occurrence of cardiovascular disease. However, it is yet unclear the value of CPET combined with RDW in predicting cardiovascular complications after thoracic surgery. 50 patients with cardiovascular complications after thoracic surgery were collected as the case group, and 100 thoracic surgery patients were recruited as the control group, with the same gender, age ± 2 years old, and no postoperative complications. After admission, all patients underwent CPET and RDW inspection before surgery, and the results were recorded. The CPET parameter oxygen pulse (VO_2_/HR) and RDW of the case group were lower than those of the control group (*P* < 0.05), and the ventilation/carbon dioxide production (VE/VCO_2_ slope) was significantly higher than control group (*P* < 0.01). The biochemical parameters hemoglobin (Hb) and Glomerular filtration rate (GFR)) of the case group were lower than those of the control group (*P* < 0.05), the homocysteine (hCY), creatinine (Cr), operation time and blood loss of the case group were higher than those of the control group (*P* < 0.05). The RDW had a negative correlation with VO_2_ max in both overall and control group. The combination of VO_2_/kg and RDW had the highest diagnostic value in predicting cardiovascular complications. The combination of VO_2_/kg and RDW has predictive diagnostic value and is more suitable for predicting postoperative complications of thoracic surgery.

## Introduction

In recent years, with the increase of aging population and the improvement of surgical techniques, major surgical procedures have been undertaken with increasing frequency^1^. More than 300 million large-scale non-cardiac surgeries are performed every year worldwide, and this number is still increasing^2^. The overall mortality rate of non-cardiac surgery complications is 0.8% to 1.5%, among them, the proportion of cardiac complications is as high as 42%^3^, whereas the incidence of cardiac death during major non-cardiac is about 0.5–1.5%^4^. Although the advantages of surgery outweigh the potential risks, the risk of perioperative complications, especially cardiovascular complications and mortality, is still a hot issue of current clinical concern. Excellent cardiopulmonary endurance is one of the necessary conditions for patients to tolerate surgery. CPET, as a noninvasive approach to assess the function of cardiopulmonary, has been proved to be a preoperative risk assessment tool to predict the occurrence of cardiovascular complications after thoracic surgery. Recent studies have shown that red blood cells (RBC) may be involved in the process of coronary vessel injury, and RDW is one of the parameters reflecting the heterogeneity of RBC size. Elevated RDW has surged as a biochemical marker in the occurrence of cardiovascular disease. In addition, there is evidence supporting a correlation between CPET parameters and RDW in patient cardiopulmonary function assessment. However, to date, no studies have combined the two indicators to predict cardiovascular complications after thoracic surgery. This study aimed to establish a new and reliable method combing the CPET parameters and RDW levels to assess the risk of cardiovascular complications after thoracic surgery.

## Materials and methods

### Patients

We retrospectively collected 600 surgical patients who had simultaneously measured data of CPET and RDW in our hospital from January 2019 to November 2020. A total of 50 patients with cardiovascular complications after thoracic surgery were recruited as the case group, and 100 patients as the control group with age ± 2years old, the same gender and surgery type, and no postoperative cardiovascular complications. The selected thoracic surgery includes esophageal surgery, mediastinal surgery, lung surgery, preventriculus surgery, etc. The participants were included in the trial regardless of the presence or absence of comorbidity such as coronary heart disease, hypertension, and diabetes. All patients are subject to strict dose control and infusion rate based on fluid intake and output. The exclusion criteria include the patients with (1) acute myocardial infarction; (2) history of the disease of gastrointestinal bleeding, anemia or other bleeding events within one year and during the follow-up period; (3) cardiac insufficiency grading III–IV with NYHA; (4) moderate and severe valvular heart disease; (5) uncontrolled cardiac arrhythmia; (6) chronic obstructive pulmonary disease (COPD); (7) concurrence of acute and chronic infectious diseases or hematological system diseases; (8) serious hepatic dysfunction or renal insufficiency; (9) concurrence of rheumatism and other autoimmune diseases; (10) hyperthyroidism or hypothyroidism; (11) a recent blood transfusion or blood donation; (12) no complete data and records. This study has been reviewed and approved by the ethics committee of the Second Affiliated Hospital of Shandong First Medical University. All methods were performed in accordance with the relevant guidelines and regulations by including a statement in the methods section.

Diagnostic criteria for cardiovascular complications after thoracic surgery: postoperative cardiovascular complications mainly refer to major adverse cardiac events that occurred during the intraoperative and postoperative period, including acute heart failure, acute myocardial infarction, severe arrhythmia, including bradyarrhythmia/ tachyarrhythmia or atrial fibrillation, and cardiogenic death^5–7^.

### Method

#### Cardiopulmonary exercise testing

CPET was performed using bicycle and pulmonary function tester (COSMED, Italy), and carried out according to standard clinical protocols. Exercise is generally divided into four stages: resting-idling-increasing load-recovery. Ramp exercise protocol: rest stage (3 min); idling without power (3 min); incrementing motion stage(5-25W/min). The total time of the incremental exercise test ranged within 10 min. A test was considered maximal if two of the following three criteria were reached: the respiratory exchange ratio (RER) ≥ 1.1, HR_max_ > 90% of the age-predicted maximum calculated according to the formula 220-age, or VO_2_ plateaued or decreased with an increase in workload. Throughout the exercise, patient's heart rate, ST segment, blood pressure, respiratory rate and other indicators were dynamically monitored. The parameters of CPET were dynamically recorded in real-time when the patient has reached the maximal exercise including MVV, VO_2_ max, VO_2_/kg, VO_2_/HR, METs, PetCO_2_, VE/VCO_2_ slope, VE/VO_2_. AT was determined using the V-slope method.

#### Laboratory testing

Fasting venous blood were sampled from each patient. All blood samples were transferred to the hospital laboratory and all tests were operated by the professional staffs. The testing indexes, such as RDW, hemoglobin (Hb), homocysteine (hCY) , total cholesterol (TC), triglyceride (TG), low density lipoprotein (LDL-C), creatinine (Cr), uric acid (UA) were obtained.

### ECG and ultrasound measures

The 12-lead ECG were performed using ECG recorder (MDS 300-4A, California, U.S) in supine position. Transthoracic echocardiography was performed to detect cardiac indexes by using ultrasound system (Philips EPIQ 7C, Amsterdam, Netherlands).

### Perioperative management

All patients received general anesthesia, the efficacy in terms of onset and duration of anesthesia and analgesia were assessed along with the heart rate, blood pressure at regular intervals throughout the perioperative period.

### Statistical analysis

The statistical software SPSS 22.0 was used for data analysis. Normal distributed variables are expressed as the mean ± standard deviation, while variables with skewed distribution are expressed as a median (interquartile range).Qualitative variables were expressed as absolute and relative frequencies. Student’s tests or the Mann–Whitney test were used for the comparison of means between two groups; Count data were compared using chi-square test. The Spearman rank correlation test was used to check correlations between the variables, and influencing factors leading to complications were analysed by logistic regression. The diagnostic value of each index was evaluated by ROC curve analysis, the value of different indicators by Delong analysis. P values less than 0.05 were considered significant.

### Consent to participate

All the patients were informed about the purposes of the study and have signed their “consent of the patient”.

## Results

### Recruitment of study participants

**Figure 1:**
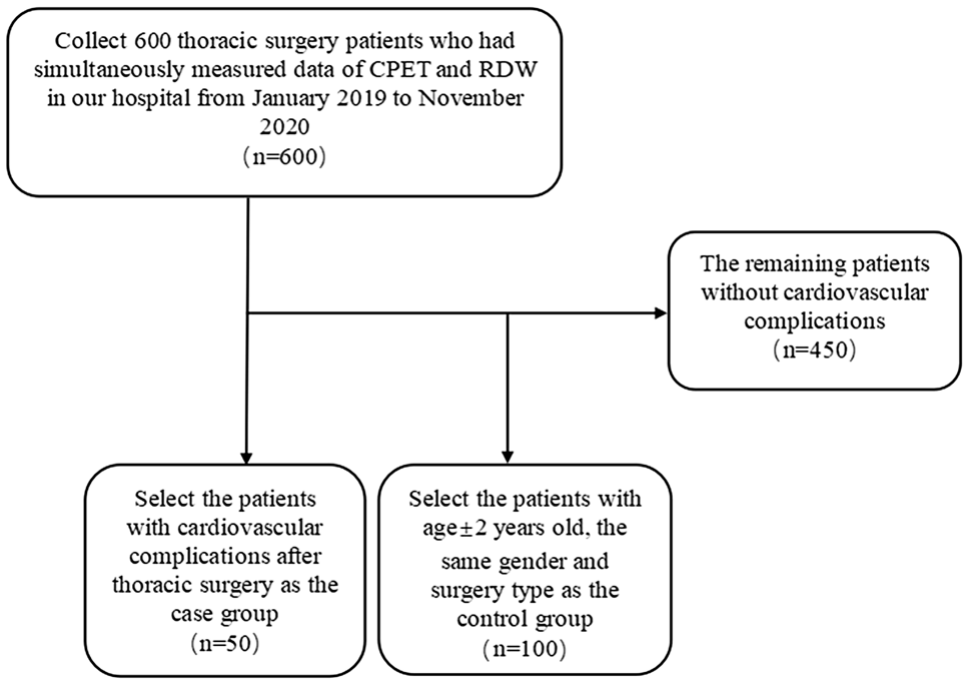
Study flow chart.

We analysed 600 patients who underwent thoracic surgery (all patients underwent therapeutic procedures) during the study period in Figure 1. We excluded 450 patients without cardiovascular complications, 120 patients did not meet criteria for maximal exercise testing. Of the 150 patients, 50 patients were included in case group and 100 patients were included in control group. Of 50 case group patients, 4 had myocardial infarction, 26 had arrhythmia, 19 had heart failure, 1 died in the postoperative period (see Fig. 1).

### General clinical data

The clinical data at baseline are shown in Table 1. Statistically significant differences for age (67.06 ± 7.52 years vs. 63.53 ± 8.17 years, *P* = 0.011), gender, smoking history were found between the groups (*P* < 0.05). There were no statistically difference between the two groups in BMI (23.82 ± 3.58 kg/m^2^ vs. 23.89 ± 3.39 kg/m^2^, *P* = 0.910), weight (66.29 ± 12.55 kg vs. 63.46 ± 10.74 kg, *P* = 0.176), comorbidities such as coronary heart disease, hypertension, and diabetes (*P* > 0.05).

**Table 1:** Comparison of clinical data between the case group and the control group.

Variable	Control group(n = 100)	Case group (n = 50)	*X*^*2*^*/t/z*	*P*
Age (years)	63.53 ± 8.17	67.06 ± 7.52	− 2.560	0.011
BMI (kg/m^2^)	23.89 ± 3.39	23.82 ± 3.58	0.013	0.910
Weight (kg)	63.46 ± 10.74	66.29 ± 12.55	1.861	0.176
Gender (%)			7.115	0.008
Male	53 (53%)	15 (30%)		
Female	47 (47%)	35 (70%)		
Smoking (%)			5.143	0.023
No	76 (76%)	29 (58.0%)		
Yes	24 (24%)	21 (42.0%)		
Hypertension (%)			1.459	0.227
No	68 (68.0%)	29 (65.0%)		
Yes	32 (32.0%)	21 (35.0%)		
Diabetes (%)			0.022	0.882
No	81 (81.0%)	41 (82.0%)		
Yes	19 (19.0%)	9 (18.0%)		
Coronary Heart Disease (%)			2.904	0.088
No	87 (87.0%)	38 (76%)		
Yes	13 (13.0%)	12 (24%)		

*P* < 0.05 was considered to be statistically significant.

### Comparison of biochemical data between case group and control group

The biochemical data are shown in Table 2. The hemoglobin (123.02 ± 16.32 g/l vs. 133.14 ± 15.02 g/l, *P* = 0.000) and GFR (92.77 ± 12.81 ml/min vs. 98.92 ± 12.69 ml/min, *P* = 0.007) of the case group were lower than those of the control group, the homocysteine (12.94 ± 4.77 umol/l vs. 10.66 ± 2.96 umol/l, *P* = 0.003) and creatinine (66.17 ± 16.78 umol/l vs. 57.52 ± 11.56 umol/l, *P* = 0.002) of the case group were higher than those of the control group, the difference were statistically significant (*P* < 0.05). There were no statistically difference between the two groups in ejection fraction (62.44 ± 4.64% vs. 63.81 ± 3.91%, *P* = 0.077), total cholesterol (4.665 (4.085, 5.59) mmol/l vs. 4.90 (4.323, 5.52) mmol/l, *P* = 0.391), triglycerides (1.035 (0.685, 1.235) mmol/l vs. 0.96 (0.763, 1.248), *P* = 0.675), low-density lipoprotein (2.768 ± 0.73 mmol/l vs. 2.714 ± 0.652 mmol/l, *P* = 0.657), and uric acid(277.5 (235.5,359.5) umol/l vs. 267.5 (206.25, 328.0) umol/l, *P* = 0.239).

**Table 2:** Comparison of biochemical data between case group and control group.

Variable	Control group (n = 100)	Case group (n = 50)	*X*^*2*^*/t/z*	*P*
Ejection fraction (%)	63.81 ± 3.91	62.44 ± 4.64	3.214	0.077
Hemoglobin (g/l)	133.14 ± 15.02	123.02 ± 16.32	13.51	0.000
Total cholesterol (mmol/l)	4.90 (4.323, 5.52)	4.665 (4.085, 5.59)	− 0.857	0.391
Triglycerides (mmol/l)	0.96 (0.763, 1.248)	1.035 (0.685, 1.235)	− 0.419	0.675
Low-density lipoprotein (mmol/l)	2.714 ± 0.652	2.768 ± 0.73	0.199	0.657
Homocysteine (umol/l)	10.66 ± 2.96	12.94 ± 4.77	9.545	0.003
Creatinine (umol/l)	57.52 ± 11.56	66.17 ± 16.78	10.751	0.002
Glomerular Filtration Rate (ml/min)	98.92 ± 12.69	92.77 ± 12.81	7.729	0.007
Uric acid (umol/l)	267.5 (206.25, 328.0)	277.5 (235.5, 359.5)	− 1.178	0.239

*P* < 0.05 was considered to be statistically significant.

### Comparison of surgical data between case group and control group

The operation time (196.0 (163.25, 270.0) min vs. 162.0 (114.25, 210.75) min, *P* = 0.000) and blood loss (60 (50.0, 100.0) ml vs. 50.0 (30.0, 100.0) ml, *P* = 0.047) of the case group were higher than those of the control group, the difference was statistically significant (*P* < 0.05). The statistical differences in other surgical data between the two groups are reported in Table 3.

**Table 3:** Comparison of surgical data between case group and control group.

Variable	Control group (n =100)	Case group (n = 50)	*X*^*2*^*/t/z*	*P*
Operation time (min)	162.0 (114.25, 210.75)	196.0 (163.25, 270.0)	− 3.638	0.000
Blood loss (ml)	50.0 (30.0, 100.0)	60 (50.0, 100.0)	− 1.98	0.047
Surgical method			0.527	0.400
Thoracotomy	23 (23.0%)	11 (22%)		
Esophagectomy	8	4		
Cardiectomy	2	1		
Mediastinal surgery	2	1		
Lobectomy	11	5		
Thoracoscopy	77 (77.0%)	39 (78%)		
Esophagectomy	16	8		
Cardiectomy	7	4		
Mediastinal surgery	5	2		
Lobectomy	49	25		
Surgery site			4.273	0.233
Esophageal	25 (25.0%)	8 (16%)		
Preventriculus	6 (6.0%)	7 (14%)		
Mediastinal	7 (7.0%)	2 (4%)		
Lung	62 (62.0%)	33 (66%)		

*P* < 0.05 was considered to be statistically significant.

### Comparative analysis of CPET index and RDW of two groups of patients before operation

The VE/VCO_2_ slop (32.36 ± 4.27 vs. 27.353 ± 2.60, *P* = 0.000) of the case group was higher than those of the control group, the VO_2_/HR (84.38 ± 13.04% vs. 106.54 ± 19.512%, *P* = 0.028) and RDW (11.938 ± 1.506% vs. 14.069 ± 1.894%, *P* = 0.000) of the case group were lower than those of the control group, the differences were statistically significant (*P* < 0.05). Statistical differences in some indicators between the two groups are reported in Table 4.

**Table 4:** Comparative analysis of CPET index and RDW of two groups of patients.

Variable	Control group(n = 100)	Case group (n = 50)	*t/z*	*p*
RDW (%)	14.069 ± 1.894	11.938 ± 1.506	6.933	0.000
MVV%pre	95.66 ± 21.07	64.42 ± 18.37	0.213	0.645
VO_2_ max%pre	91.33 ± 13.62	67.70 ± 13.13	0.016	0.890
VO_2_/kg (ml/min/kg)	21.41 ± 3.656	15.98 ± 2.97	0.70	0.404
VO_2_/HR (%)	106.54 ± 19.512	84.38 ± 13.04	4.914	0.028
METs	6.213 ± 1.00	4.59 ± 0.87	0.018	0.893
AT (ml/min/kg)	17.08 ± 2.83	12.07 ± 2.28	1.89	0.172
PetCO_2_ (mmHg)	37.28 ± 5.05	36.60 ± 5.17	0.586	0.446
VE/VO_2_	35.16 ± 6.31	34.12 ± 5.57	1.06	0.305
VE/VCO_2_ slop	27.353 ± 2.60	32.36 ± 4.27	17.162	0.000

*P* < 0.05 was considered to be statistically significant.

### Multivariate logistic regression analysis of postoperative cardiovascular complications

With the occurrence of postoperative cardiovascular complications as the dependent variable, 8 indicators including operation time, hypertension, RDW, VO_2_/kg, AT, VO_2_ max, MVV, and METs were used as independent variables. The multi-actor unconditional logistic regression analysis revealed that the differences in all indicators were not statistically significant (*P* > 0.05) (see Table 5).

**Table5:** Logistic regression analysis of postoperative cardiovascular complications.

Variable	*B*	*S.E*	*Wald*	*p*	OR	95% CI	
						Lower limit	Upper limit
Operation time	0.039	0.029	1.791	0.181	1.040	0.982	1.101
Hypertension	− 3.917	2.623	2.231	0.135	0.020	0.000	3.399
RDW (%)	− 5.719	3.349	2.917	0.088	0.003	0.000	2.326
MVV%pre	− 0.252	0.132	3.675	0.055	0.777	0.600	1.006
VO_2_ max%pre	− 0.136	0.087	2.461	0.117	0.873	0.737	1.034
VO_2_/kg	− 1.121	0.976	1.318	0.251	0.326	0.048	2.209
METs	4.296	3.578	1.441	0.230	73.394	0.066	81,551.255
AT	− 3.616	2.061	3.080	0.079	0.027	0.000	1.526

*P* < 0.05 was considered to be statistically significant.

### Correlation analysis of CPET index and RDW in patients undergoing thoracic surgery

Spearman rank correlation test was used to analyze the correlation between RDW and other variables. The results showed that RDW had a negative correlation with VO_2_ max in overall. RDW had a negative correlation with VO_2_ max in control group. There was no correlation between RDW and each index in the case group (see Table 6).

**Table 6:** Correlation analysis of thoracic surgery patients and their components.

	RDW (%)					
	Overall*		Control group^**△**^		Case group^#^	
	*r*	*p*	*r*	*p*	*r*	*p*
MVV%pre	− 0.070	394	0.158	0.116	− 208	0.147
VO_2_ max%pre	− 0.207	0.011	− 0.092	0.364	− 0.277	0.052
VO_2_/kg(ml/min/kg)	− 0.110	0.181	0.016	0.871	− 0.142	0.3265
VO_2_/HR%	− 0.156	0.057	− 0.089	0.376	− 0.170	0.237
METs	− 0.105	0.202	0.022	0.830	− 0.118	0.416
AT(ml/min/kg)	− 0.143	0.081	− 0.049	0.630	− 0.142	0.324
PetCO_2_(mmHg)	− 0.020	0.812	− 0.208	0.037	0.220	0.125
VE/VO_2_	− 0.018	0.831	0.051	0.611	− 0.089	0.537
VE/VCO_2_slop	− 0.029	0.727	0.023	0.823	− 0.239	0.095

*Total number of thoracic surgery, ^△^Uncomplicated group, ^#^Complication group.

### Evaluation of diagnostic value of CPET indexes

**Figure 2:**
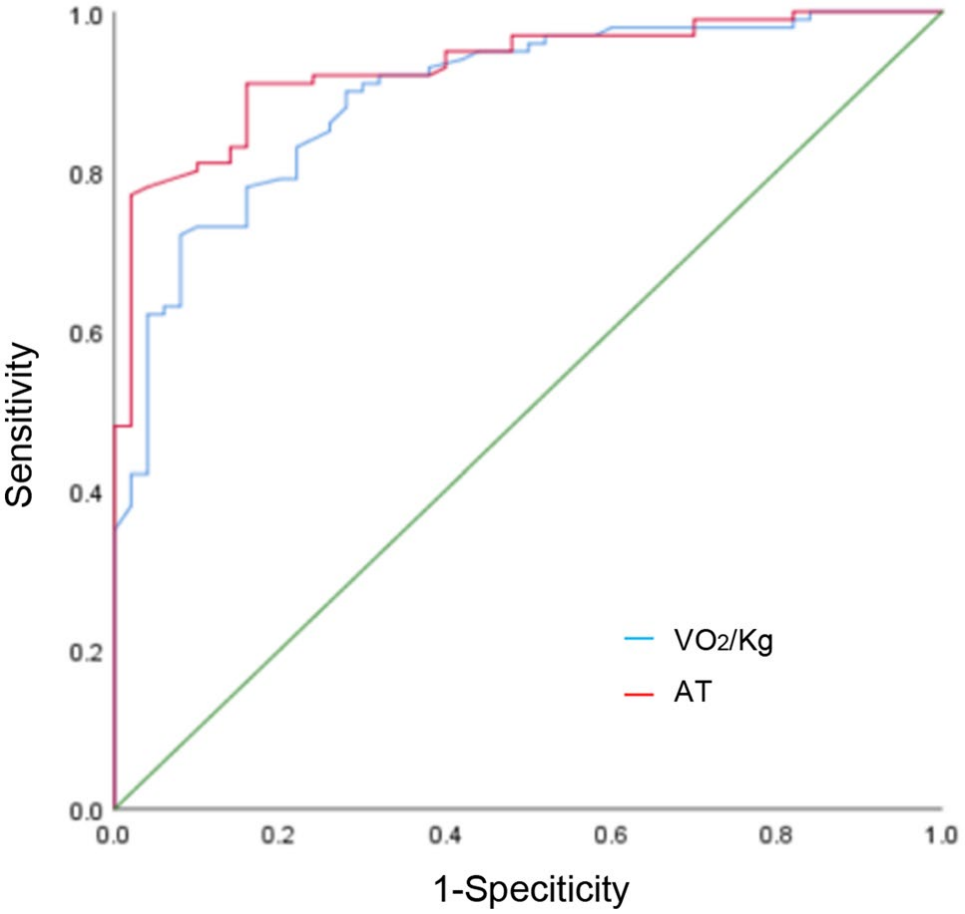
The ROC curve of some indicators of CPET for predicting cardiovascular complications after thoracic surgery.

The ROC curve analysis showed that the indicators including VO_2_/kg and AT had high diagnostic value, and the area under the curve (AUC) of AT was higher (AUC = 0.930, 95% CI 0.899–0.964, *P* < 0.01) than that of VO_2_/kg (AUC = 0.894, 95% CI 0.843–0.946, *P* < 0.01). Meanwhile, Delong analysis showed that the difference was statistically significant, suggesting that AT had a higher diagnostic value (see Fig. 2).

### Evaluation of the diagnostic value of RDW, VO_2_/kg and the combination of the two

**Figure 3:**
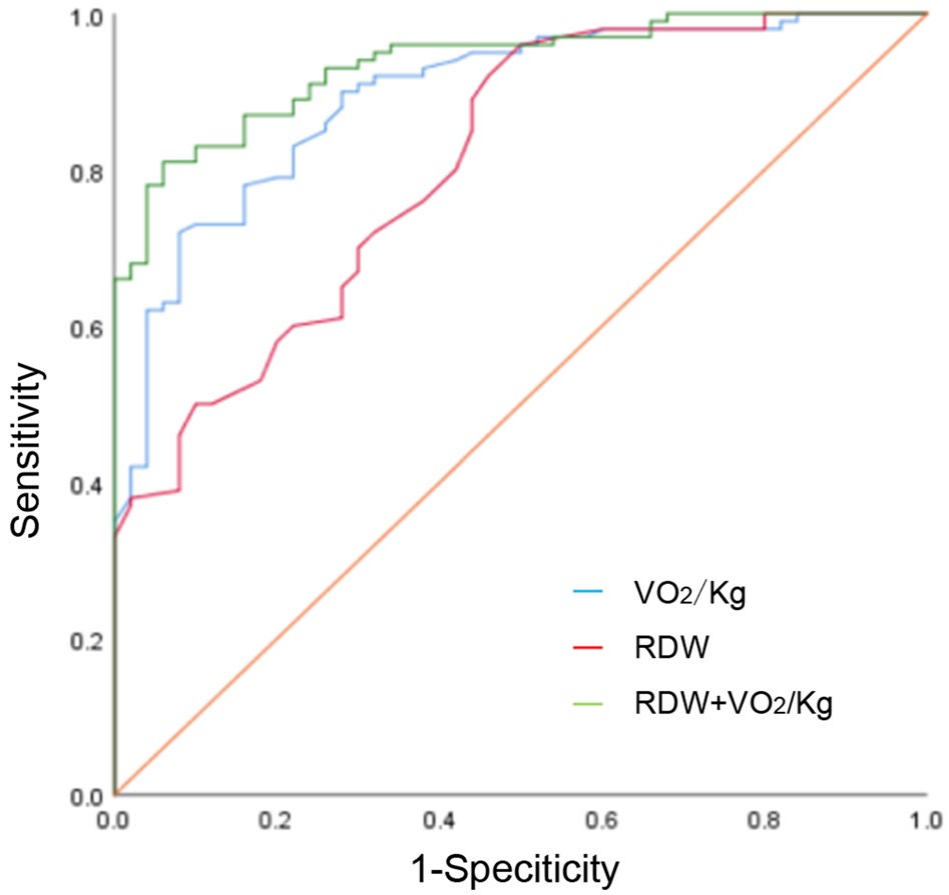
The ROC curve of VO_2_/kg, RDW and the combination of the two for predicting postoperative cardiovascular complications.

The two indicators were combined into a new indicator through Logistic regression, and the ROC curve predictive value of the new indicator was evaluated. The area under the VO_2_/kg curve was 0.894 (95% confidence interval: 0.843–0.946, *P* < 0.01), and the area under the RDW curve was 0.808 (95% confidence interval: 0.736–0.880,* P* < 0.01). The area under the combined curve of the two indicators is 0.936 (95% confidence interval: 0.900–0.972, *P* < 0.01). Delong analysis showed that the difference in RDW vs VO_2_/kg, RDW vs combined diagnosis index was statistically significant. It showed that RDW had the relatively lower diagnostic value, meanwhile, the difference in VO_2_/kg vs combined diagnosis index was not statistically significant, indicating that these two indicators had high diagnostic value. However, the combined diagnostic index had the larger AUC and the sensitivity and specificity were both high, therefore, it had more advantages than a single index in predicting and diagnosing postoperative cardiovascular complications (see Fig. 3).

## Discussion

Although surgical operations can improve the quality of life and prolong life expectancy of patients, the occurrence and severity of postoperative complications cannot be ignored, especially cardiovascular complications. Postoperative cardiovascular complications are the main cause of postoperative death for patients, it can directly affect the recovery of postoperative patients, determine the success or failure of the operation, and even endanger the patient’s life.2014 ESC/ESA Guidelines on Non-cardiac Surgery: Cardiovascular Assessment and Management showed that cardiac complications after non-cardiac surgery originate from patient-related risk factors, the type of surgery, and the circumstances under which it takes place^8^. 2014 ACC/AHA Guideline showed that the patient’s cardiopulmonary function is the crux of the preoperative cardiac risk assessment. Poor cardiopulmonary function is related to the increased incidence of postoperative cardiac adverse events^9^. The operation puts a tremendous load on the patient’s cardiorespiratory reserve, increasing the oxygen demand by nearly 40%^10^. Due to the limitations of medical technology and conditions in the past, the accuracy of evaluating preoperative function and predicting complications or death after surgery was poor^11,12^, therefore, more accurate alternatives are needed to evaluate preoperative functional ability and then predict the outcome of surgery. Cardiopulmonary exercise testing (CPET) is a useful and non-invasive tool to objectively and safely evaluate the cardiopulmonary function and exercise capacity^13^, as a tool for evaluating exercise endurance, it can dynamically conduct a comprehensive and objective comprehensive response to incremental exercise, accurate analysis and assessment of individualized cardiorespiratory reserve capacity and functional impairment to determine the operability of the operation, judge the risk stratification and prognosis.

First, CPET imposes a load on the heart–lung-oxygen delivery system to increase the patient’s ventilation, oxygen uptake, carbon dioxide production, cardiac output, and pulse rate, it can simulate the load exerted on the recipient during the operation, and evaluate the patient's tolerance to the operation comprehensively. Secondly, patients are often stressed during the perioperative period, and many patients are in a compensatory state of cardiopulmonary function under static conditions, which indicators cannot accurately determine the prognosis. If the intraoperative and postoperative treatment strategies are formulated according to conventional parameters, it may increase the risk of perioperative complications. As the gold standard for assessment of cardiopulmonary function, CPET can comprehensively assess the cardiovascular, respiratory, muscular and metabolic systems during exercise. Patients with high-risk static lung function assessment were thought to be less able to tolerate surgery, such as forced expiratory volume in the first second before surgery (FEV1) as a percentage of the predicted value ≤ 40% of the predicted value, PaCO_2_ ≥ 45 mmHg (1 mmHg = 0.133 kPa), etc. Morice et al.^14^ conducted exercise tests on 37 patients who were considered ineligible for surgery according to conventional pulmonary function criteria, it was proved that preoperative CPET assessment is more objective and reliable than ordinary pulmonary function tests.

Among numerous studies on CPET, VO_2_ max and AT are commonly used to assess the severity of the disease and evaluate the patient's cardiopulmonary function reserve, reflecting the degree of impaired oxygen delivery capacity of the circulatory system and respiratory capacity. VO_2_ max is jointly affected by the heart reserve function and muscle uptake and oxygen consumption. It represents the ability of the blood and circulatory system to transport oxygen. Exercise endurance depends more on the oxidative capacities of the muscle, i.e., on mitochondrial metabolism, and is closely related to AT. But in heart failure (HF) patients, during maximal CPET, AT is not always identified^15^. In addition, VO_2_ max depends on the function of the circulatory and respiratory system, and is also affected by body shape, muscle mass and health status; In order to regulate the VO_2_ between individuals of different body types to better reflect the relative fitness, it is usually expressed in the form of VO_2_/kg.

RDW is a parameter that reflects the heterogeneity of red blood cell volume^16^. Recent studies have found that RDW may be a marker for cardiac risk stratification and outcomes evaluation for coronary heart disease^17–19^. Studies have shown that RDW is closely related to chronic subclinical inflammation, oxidative stress, malnutrition, serum C-reactive protein, interleukin-6, erythrocyte sedimentation rate and natriuretic peptide^20–22^. Inflammation is the key to the occurrence and intensification of vascular disease^23^, meanwhile surgery induces the production of pro-inflammatory cytokines to higher inflammation activation in patients during surgery^24^. Tonelli et al.^18^ reported that increased RDW is independently associated with cardiovascular events and the risk of all-cause death. This association may be mediated through chronic inflammation which can be used to improve the effectiveness of risk stratification and monitoring risk adjustment strategies for patients before surgery. We have found that there is a close correlation between patients with low preoperative RDW and poor long-term survival.

In order to find an easy and reliable method to estimate the risk and prognosis of patients with thoracic surgery, we introduce RDW on the basis of CPET research. The CPET parameter oxygen pulse (VO_2_/HR) and RDW of the case group were lower than those of the control group (*P* < 0.05), and the ventilation/carbon dioxide production VE/VCO_2_ slope) was significantly higher than control grou The RDW of the control group was significantly greater than that of the case group, and the difference was statistically significant. When we further combined VO_2_/kg and RDW, we found that the combined diagnosis has the largest area under the ROC curve and the sensitivity and specificity are more advantageous than a single index.

Glowczynska et al.^25^ confirmed that higher RDW is independently related to peakVO_2_ and VE/VCO_2_ slope only in patients with chronic heart failure (CHF), and it may be a sign of further aggravation of HF. Nishiyama et al.^26^ proved that significant inverse correlations were observed between peak VO_2_ and RDW. Elevated RDW reflects a reduction in oxygen transport capacity which may be one of the mechanisms responsible for the relationship between increased RDW and poor prognosis in cardiovascular disease, exercise training increases exercise tolerance and decreases RDW in association with increased oxygen uptake in patients with CAD. This is similar to our conclusion that there is a correlation between RDW and CPET, which supports the correlation between RDW and cardiopulmonary endurance. Our results are in contrast to the above mentioned studies, it may be due to an insufficient sample size.

So far, there are few studies on the correlation between RDW and CPET and their combined application in cardiovascular complications after thoracic surgery. On the basis of predecessors, this research innovatively proposes the combined application of the two in disease diagnosis, it improves the diagnostic basis for thoracic surgery complications, especially cardiovascular complications. With the increase of new indications for CPET and RDW, it may also provide a certain value for the diagnosis and prevention of other diseases. This is the shortcoming of the existing literature.

Due to a case–control study, there are limitations related to this study such as selection bias, uncertain causality, etc. In addition, the sample size is relatively small, and no subgroup analysis was performed. So, more studies are needed to verify this conclusion.

## Data Availability

Data and material are transparent, and the corresponding author can be contacted if requested.
